# Complete genome sequence of *Chlamydia psittaci* АМК-16, isolated from a small ruminant in the Middle Volga Region, Russia

**DOI:** 10.1128/mra.00543-23

**Published:** 2024-03-27

**Authors:** Sergey Zaitsev, Mariya Khizhnyakova, Yury Saltykov, Vitaliy Evstifeev, Fidail Khusainov, Svetlana Ivanova, Daria Morozova, Sergey Yakovlev, Olga Larionova, Valentina Feodorova

**Affiliations:** 1 Saratov State University of Genetics, Biotechnology and Engineering named after N.I. Vavilov, Saratov, Russia; 2 Federal Center for Toxicological, Radiation and Biological Safety, Kazan, Russia; 3 Kazan State Academy of Veterinary Medicine by N.E. Bauman, Kazan, Russia; Loyola University Chicago, Chicago, Illinois, USA

**Keywords:** chlamydia, whole-genome, SNP, АМК-16, small ruminants, goat, abortion, chlamydia infection, livestock

## Abstract

We report the complete genome sequence of the *Chlamydia psittaci* АМК-16, recovered from the aborted caprine fetus during a case of chlamydia infection. This 1,152,497-bp genome with 7,552-bp cryptic plasmid provides novel insights into the genetic diversity of chlamydia agent strains particularly those causing the infection in small ruminants.

## ANNOUNCEMENT

Infectious abortions in small ruminants are generally associated with *Chlamydia abortus* and rarely with other *Chlamydia* spp. (1–12). Currently, there are only few reports on *Chlamydia psittaci,* an obligate intracellular Gram-negative bacterium, as the causative agent of abortion in small ruminants, goats, and sheep (1, 8, 13, 14) (https://goats.extension.org/chlamydiosis/, https://www.oie.int/fileadmin/Home/eng/Health_standards/tahm/3.08.05_ENZ_ABOR.pdf). In this paper, we report the complete genome sequence of the *C. psittaci* strain АМК−-16, isolated from the embryonated egg yolk sacs inoculated with fetal organ suspensions derived offrom an aborted dairy goat during a local chlamydia outbreak in the Middle Volga Region, Russia, in 2016. The strain was purified from contaminating substances through Renografin density gradient centrifugation of the egg-derived chlamydiae suspension (15). The DNA was extracted from the lyophilized suspension of the strain, using a commercial DNA DNeasy Blood & Tissue Kit (Qiagen, Germany). The final DNA concentration was measured spectrophotometrically (Bio-Rad, USA) for following parallel sequencing using two platforms, Illumina and Nanopore. Preparation of DNA libraries for sequencing was performed using: (i) Nextera XT DNA Library Preparation Kit (illumine Inc., USA) or (ii) Nanopore Kit SQK-LSK109 (Oxford Nanopore, UK) without prior DNA shearing, respectively.

Totally, there were 6,884,924 single reads of 250 bp length obtained using the Illumina platform (Illumina Inc., USA) and 1,472,000 reads generated by MinION (Oxford Nanopore, UK) sequencing (N_50_ = 12,901). Evaluation and quality control of short reads were performed using FastQC software v0.11.9 (16). Filtering, trimming, and deleting the adapter sequences in the “raw” data array from the platform Illumina was carried out using AfterQC v0.9.7 (17). Long reads were filtered using Filtlong v0.2.0 with a minimum cutoff of 2,000 bp. Taxonomic classification was carried out using raw reads by with Kraken v2 (18). The *de novo* hybrid assembly was done using the Unicycler assembler v0.4.1 (19). Default parameters were used unless otherwise stated. Two different circular contigs (coverage, 150X×) were generated. The largest contig of 1,152,497 bp was associated with the AMK-16 strain chromosome, while the smaller contig of 7,552 bp corresponded to the extrachromosomal cryptic plasmid. The G + C contents were 39.12% and 32.9%, respectively. Genome annotation was performed with the NCBI Prokaryotic Genome Annotation Pipeline (PGAP) v4.10 (20) at the National Center for Biotechnology Information (NCBI). Further, 981 CDSs were identified in the strain chromosome and 8 CDSs in the plasmid. Based on Similar Genome Finder Service of BV-BRC platform v3.28.22 (https://www.bv-brc.org) (21), the plasmid-bearing *C. psittaci* Rostinovo-70 strain (GenBank accession number CP041038.1) which previously isolated from the aborted livestock pathological material (15) was assigned as the highest homolog for the AMK-16 (distance value 0). Pairwise comparative analysis of the AMK-16 and Rostinovo-70 chromosomes using Mauve v2015.02.26 (22) revealed 86 SNPs, demonstrating no absolute identity between these strains. The plasmidless *C. psittaci* GR9 strain (23) (GenBank accession number CP003791.1) derived from the duck was identified as another additional homolog for the AMK-16 (distance value 0.0011856). Generally, 1,967 SNPs were found in the relevant chromosomes.

Thus, the *C. psittaci* AMK-16 is the second strain after the Rostinovo-70 (15), which was isolated in Russia from small ruminants with chlamydia infection.

## Data Availability

The *C. psittaci* АМК-16 genome sequences are available in GenBank under accession numbers: CP047319.1 (for the chromosome) and CP047320.1 (for the plasmid pAMK). BioSample number SAMN13684682, and BioProject number PRJNA597844. SRA accession numbers were SRX19561946 for Oxford Nanopore data and SRX19561945 for Illumina HiSeq2500 data.
